# Genome-Wide Identification and Expression Analysis of the COL Gene Family in *Hemerocallis citrina* Baroni

**DOI:** 10.3390/cimb46080503

**Published:** 2024-08-05

**Authors:** Ziwei Zuo, Guangying Ma, Lupeng Xie, Xingda Yao, Shuxia Zhan, Yuan Zhou

**Affiliations:** Zhejiang Institute of Landscape Plants and Flowers, Hangzhou 311251, China; ziweizuo@yeah.net (Z.Z.); xielupeng@foxmail.com (L.X.); yaoxingda1995@163.com (X.Y.); zhanpangxie@163.com (S.Z.); dazhouyuan19@163.com (Y.Z.)

**Keywords:** COL genes, phylogenetic relationship, collinearity, gene expression, *Hemerocallis citrina*

## Abstract

*Hemerocallis citrina* Baroni (*H*. *citrina*) is an important specialty vegetable that is not only edible and medicinal but also has ornamental value. However, much remains unknown about the regulatory mechanisms associated with the growth, development, and flowering rhythm of this plant. CO, as a core regulatory factor in the photoperiod pathway, coordinates light and circadian clock inputs to transmit flowering signals. We identified 18 COL genes (*HcCOL1*-*HcCOL18*) in the *H. citrina* cultivar ‘Mengzihua’ and studied their chromosomal distribution, phylogenetic relationships, gene and protein structures, collinearity, and expression levels in the floral organs at four developmental stages. The results indicate that these genes can be classified into three groups based on phylogenetic analysis. The major expansion of the HcCOL gene family occurred via segmental duplication, and the Ka/Ks ratio indicated that the COL genes of *Arabidopsis thaliana*, *Oryza sativa*, *Phalaenopsis equestris*, and *H*. *citrina* were under purifying selection. Many *cis*-elements, including light response elements, abiotic stress elements, and plant hormone-inducible elements, were distributed in the promoter sequences of the HcCOL genes. Expression analysis of HcCOL genes at four floral developmental stages revealed that most of the HcCOL genes were expressed in floral organs and might be involved in the growth, development, and senescence of the floral organs of *H*. *citrina*. This study lays a foundation for the further elucidation of the function of the HcCOL gene in *H. citrina* and provides a theoretical basis for the molecular design breeding of *H. citrina*.

## 1. Introduction

*Hemerocallis citrina* Baroni (*H*. *citrina*, also called the yellow flower vegetable in China) is a perennial rooted herbaceous plant in the *Hemerocallis* genus of the *Asphodelaceae* family. It is valued for its edible and medicinal properties, as well as its ornamental value. The main cultivated areas for this plant in China include Datong (Shanxi), Qingyang (Gansu), Dali (Shaanxi), Qidong (Hunan), and Jinyun (Zhejiang). The edible parts of *H*. *citrina* are the flower buds, which are harvested before flowering. The timing of plant flowering is a crucial biological characteristic that is related to the reproductive capacity and adaptability of the plant. *H*. *citrina* is a typical LD plant whose flowering phase occurs in mid-to-late May each year. The flowering period of a single flower is approximately 12 h, and most species exhibit flowering in the evening or at night and closing during the day, with distinct rhythmic characteristics. Currently, the mechanisms regulating the growth, development, and flowering rhythm of *H. citrina* are not fully understood. Given the demand for flowering regulation, market value enhancement, and ornamental quality improvement for this plant, the relevant molecular mechanisms urgently need to be investigated.

In plants, precise regulation of flowering time is crucial for successful reproduction, and flowering is triggered by endogenous pathways and environmental cues. At least six flowering pathways exist in higher plants: the photoperiod pathway, the vernalization pathway, the ambient temperature pathway, the autonomous pathway, the gibberellin pathway, and the age pathway [1].

CONSTANS (CO) is the key component of the photoperiodic pathway, functioning as a transcription factor that can directly bind to DNA to activate the transcription of its target gene [2]. CO was discovered due to the late-flowering phenotypes exhibited by CO mutant plants [3,4]. In addition, the overexpression of CO leads to early flowering [5]. CO encodes a protein containing two conserved segments and one or two N-terminal B-box zinc-finger domains, which are predicted to mediate protein-protein interactions, along with a C-terminal CCT domain for nuclear localization and to mediate protein-protein interactions; the activation of downstream genes is facilitated by CO, which may be recruited to promoters by DNA-binding proteins [6]. CO promotes flowering in *Arabidopsis* by activating SUPPRESSOR OF OVEREXPRESSION OF CONSTANS 1 (SOC1) through FLOWERING LOCUS T (FT), with FT being necessary for the activation of SOC1 by CO [7]. Subsequent research has indicated that the B-box of CO facilitates oligomerization, while the C-terminal CCT domain plays a role in the formation of the CO-CCT-NF-Y complex. This complex then binds to the four TGTG motifs in the FT promoter, mediating FT activation [8].

The *Arabidopsis* COL family contains 17 genes, the majority of which are functionally linked to the regulation of flowering, while some are involved in other regulatory functions. Constitutive expressions of *COL1* and *COL2* have no significant effects on flowering time [9]. The overexpression of *COL5* leads to early flowering under short-day (SD) conditions, and a *COL5* loss-of-function mutation does not affect the flowering time [10]. *COL8*-overexpressing plants exhibit a late-flowering phenotype under long-day (LD) conditions [11]. COL9 and COL10 act as negative regulators of flowering in the photoperiodic pathway, where COL9 may affect flowering time by repressing the expression of *CO* and simultaneously reducing the expression of *FT* [12]. The overexpression of *COL12* inhibits *FT* expression, and COL12 inhibits flowering by inhibiting CO protein function through interactions with CO [13]. COL4 functions as a flowering repressor under LD and SD conditions [14]. Certain COL genes not only participate in the regulation of flowering but also perform other functions. CO, a key regulator of photoperiodic flowering, also participates in promoting flower senescence and abscission by enhancing JA signaling and response [15]. Under LD conditions, CO interacts with key transcription factors, ABFs, in the ABA signaling pathway to produce antagonistic effects, thus impeding plant tolerance to salt stress [16]. CO directly inhibits AP2 transcription, regulates seed size by controlling the proliferation of seed coat epidermal cells, and functions in a maternal-dependent manner [17]. The transcription factor COL4 in *Arabidopsis* acts as a flowering repressor and promotes abiotic stress tolerance in a gibberellic acid-dependent manner [18]. Some COL genes are involved in the regulation of vegetative organ development. COL3, which promotes meristem formation and inhibits the growth of primary shoots under SD conditions, acts downstream of CONSTITUTIVE PHOTOMORPHOGENIC1 (COP1), an E3 ubiquitin ligase that suppresses photomorphogenesis in darkness; COL3 can promote lateral root development independently of COP1 [19]. COL7 promotes branching under conditions with a high red light: far-red light ratio (R:FR) but enhances shade avoidance syndrome under low-R:FR conditions [20]. Disrupting the function of COL13 by T-DNA insertion or RNAi results in the formation of longer hypocotyls in *Arabidopsis* seedlings under red light, and conversely, the overexpression of *COL13* results in the formation of shorter hypocotyls [21].

There are 16 members of the rice COL gene family, and *Heading date 1* (*Hd1*, *OSA*) is a homologue of *CO* in rice that also plays a photoperiodic role. *Hd1* has a primary function for promoting the expression of *Heading date 3a* (*Hd3a*)/*RICE FLOWERING LOCUS T 1* (*RFT1*), regardless of the day-length, as photoperiodic flowering in rice is regulated by the crosstalk of two modules: Hd1 promotes *Grain number*, *plant height*, and *heading date 7* (*Ghd7*) expression and is recruited by Ghd7 and/or Days to heading on chromosome 8 (DTH8) to form repressive complexes that collaboratively suppress the *Early heading-date 1 (Ehd1)–Hd3a*/*RFT1* pathway in order to repress the heading under LD conditions and under SD conditions; owing to the weakened suppression effect of Ghd7, the repressive functions of these complexes are decreased, and Hd1 competes with the complexes to promote *Hd3a*/*RFT1* expression, playing a tradeoff relationship with photoperiod sensitivity flowering [22,23]. OsCO3 (OsB) is a negative regulator of the photoperiodic regulation of flowering under SD conditions, and OsCO3 controls flowering time mainly by negatively regulating the expressions of *Hd3a* and *Flowering Locus T-like* (*FTL*), independent of the SD promotion pathway [24]. OsCOL10 (OsJ) suppresses flowering by decreasing the expressions of *RFT1* and *Hd3a* through *Ehd1* [25]. *Grain number*, *plant height*, and *heading date 2* (*Ghd2*, *OsK*) play important roles in accelerating drought-induced leaf senescence in rice [26]. OsCOL15 (OsO) suppresses flowering by upregulating *Ghd7* and downregulating the flowering activator *Rice Indeterminate* 1 (*RID1*), resulting in the downregulation of the flowering activators *Ehd1*, *Hd3a,* and *RFT1* [27]. OsCOL16 (OsL) upregulates the expression of the flowering inhibitor *Ghd7*, leading to the downregulation of the expressions of *Ehd1*, *Hd3a*, and *RFT1*, thereby inhibiting rice heading [28].

In addition, COL genes have been identified in other plants. GmCOL1a enhances salt and drought tolerances in transgenic soybean plants by promoting GmP5CS protein accumulation in hairy roots [29]. Potato plants may contain two CO/FT modules: one that controls tuber induction according to day length and another that influences day-neutral flowering [30]. In mango, MiCOL2 and MiCOL9 negatively regulate flowering time and positively regulate tolerances to salt and drought stresses [31,32]. MaCOL1 in banana may be involved in fruit ripening and stress responses [33]. In *Phyllostachys edulis*, most *PheCOLs* likely play a role in the stem maturation process [34]. Thus, COL genes have a wide range of effects on plant development.

The COL gene family has been extensively studied in the model plants *Arabidopsis* and rice, but previous research focused primarily on floral transition; there have been few studies on the impact of CO on the opening and closing of a single flower. However, sufficient research on the function and regulatory mechanism of the COL family in *H*. *citrina,* an important specialty perennial vegetable, is lacking. Therefore, the study of the function and regulatory mechanism of the COL gene family will help to reveal the molecular mechanism of flower bud growth and development and stress resistance regulation in *H*. *citrina*, which is of great practical significance for the cultivation of new and superior varieties that exhibit high resistance, daytime blooming, and off-season blooming.

## 2. Materials and Methods

### 2.1. Plant Material and Sample Collection

The *H. citrina* cultivar ‘Mengzihua’ was grown in the experimental field of the Zhejiang Institute of Landscape Plants and Flowers, Hangzhou, China. To investigate changes in COL gene expression during different developmental stages of *H. citrina* flowers, flower organs at four developmental stages, namely young flower buds (F1), flower buds (F2), blooming flowers (F3), and spent flowers (F4), were selected for sampling. The young flower buds showed the embryonic form of buds, were approximately 3.5 cm in size, and were sampled at 9 a.m.; the flower bud stage was the harvesting period for *H. citrina*, and the flower buds were close to fully yellow, were approximately 15 cm in size, were in an unopened state, and were sampled at 9 a.m.; at the blooming stage, the flowers were in full bloom, with fully expanded petals, and were sampled at 8 p.m.; in the spent flower stage, the flowers were decayed and folded, and sampling was performed at 9 a.m. on the day following the opening of the flowers. All samples were immediately frozen in liquid nitrogen after harvest and stored at −80 °C until further use.

### 2.2. Identification, Distribution, and Physicochemical Analysis of H. citrina COL Genes

The whole-genome sequences of *H. citrina* and *Phalaenopsis equestris* (*P*. *equestris*) were downloaded from NCBI (https://www.ncbi.nlm.nih.gov/genome/, accessed on 20 March 2023), and those of *Arabidopsis thaliana* (*A. thaliana*) and *Oryza sativa* (*O*. *sativa*) were obtained from TAIR (http://www.Arabidopsis.org/, accessed on 20 March 2023) and MSU (http://rice.uga.edu/, accessed on 20 March 2023), respectively. The COL amino acid sequences of *A. thaliana*, *O*. *sativa,* and *P*. *equestris* were used as seed sequences, and TBtools (version v1.31) [35] was used to perform BLAST (E-value < 10^−10^) against the *H. citrina* genome. Three hidden Markov models (HMMs) were used to screen the amino acid sequences after BLAST for identification: COL10 (PTHR31717), B-box (PF00643), and CCT (PF06203) (https://www.ebi.ac.uk/interpro/, accessed on 29 March 2023). The candidate genes were screened based on the models using HMMER 3.0 (version 3.3.2) (E-value < 10^−5^) [36], and proteins satisfying all three models were identified as candidate COL family members. Candidate genes were verified using the Conserved Domain Database (CDD) (https://www.ncbi.nlm.nih.gov/Structure/cdd/, accessed on 2 April 2023) and the Simple Modular Architecture Research Tool (SMART) (http://smart.embl-heidelberg.de, accessed on 2 April 2023). Finally, the HcCOL genes were identified after a comprehensive curation of the *H. citrina* genome. All the COL genes from the four species (*A. thaliana*, *O. sativa*, *P*. *equestris,* and *H. citrina*) are shown in Supplementary Table S1. The distributions of HcCOL genes on the chromosomes were determined using TBtools (version v1.31) [35]. The HcCOL gene information was obtained from the GFF file. The basic physicochemical properties of the HcCOL protein were analyzed using ProtParam (https://web.expasy.org/protparam/, accessed on 10 April 2023). Subcellular locations were predicted by Plant-mPLoc (http://www.csbio.sjtu.edu.cn/bioinf/plant-multi/, accessed on 10 April 2023).

### 2.3. Gene Structure, Conserved Domain Analyses, and Three-Dimensional Models of the B-Box

Exon-intron information for the HcCOL gene was obtained from GFF files, and the conserved domains were annotated using the CDD (https://www.ncbi.nlm.nih.gov/Structure/cdd/, accessed on 2 April 2023) and displayed using TBtools (version v1.31) [35]. Additionally, the three-dimensional models of the conserved B-box domains of the HcCOL proteins were predicted by Phyre2 (http://www.sbg.bio.ic.ac.uk/phyre2/html/, accessed on 10 April 2023).

### 2.4. Sequence Alignment, Phylogenetic Analysis, and Collinearity Analysis of HcCOLs from H. citrina and Other Plants

Needle (https://www.ebi.ac.uk/Tools/psa/emboss_needle/, accessed on 23 April 2023) was used to perform the pairwise alignment of protein sequences to determine the similarity and identity between COL members. Multiple sequence alignments were performed by ClustalW (http://www.genome.jp/tools/clustalw/, accessed on 23 April 2023), and Jalview (version 2.11.2.0) [37] was utilized to highlight conserved or similar amino acid sequences. A phylogenetic tree was constructed by the neighbor-joining method using MEGA X [38] (JTT mode, pairwise deletion, and 1000 bootstrap values) and was visualized using iTOL (https://itol.embl.de/, accessed on 5 June 2023). Collinearity analysis among the HcCOL members and synteny analysis between *H. citrina* and *A. thaliana*, *O. sativa*, and *P. equestris* were performed using TBtools (version v1.31) [35]. The Ka (non-synonymous substitution rate) and Ks (synonymous substitution rate) values were calculated by TBtools (version v1.31) [35].

### 2.5. Analysis of Cis-Acting Elements

A 2000 bp sequence upstream of the ATG initiation codon of the HcCOL gene was extracted and submitted to PlantCARE (http://bioinformatics.psb.ugent.be/webtools/plantcare/html/, accessed on 5 June 2023) to predict *cis*-acting elements in the promoter region, after which selection, statistical analysis, and mapping were conducted.

### 2.6. Expression Analysis by qRT-PCR

Total RNA of F1-F4 was extracted using an RNAprep Pure Plant Kit (TIANGEN, Beijing, China) according to the manufacturer’s instructions. Genomic DNA was removed using DNase I. RNA integrity, concentration, and purity were analyzed via NanoDrop 2000 (Thermo Fisher, Waltham, MA, USA) and 1.5% agarose gel electrophoresis. First-strand cDNA was synthesized by 1 μg of RNA using a ReverTra Ace-α-^®^ Kit (TOYOBO, Osaka, Japan), and qRT-PCR analyses were performed using a Step One Plus^TM^ Real-Time PCR Instrument Thermal Cycling Block (Thermo Fisher, Waltham, MA, USA) with 20 μL of reaction volume, which was comprised of 10 μL of PowerUp™ SYBR™ Green Master Mix (Thermo Fisher, Waltham, MA, USA), 1 μL of forward primers (10 μM), 1 μL of reverse primers (10 μM), 1 μL of cDNA (50 ng/μL), and 7 μL of RNase-free ddH_2_O. The PCR thermocycling program used was as follows: the initial uracil-DNAglycosylase step at 50 °C for 2 min; pre-denaturation at 95 °C for 2 min; denaturation at 95 °C for 15 s; annealing at 55 °C for 15 s; and extension at 72 °C for 1 min for 40 cycles. *HcAP4* was used as an internal control. The relative gene expression levels were calculated by the 2^−ΔΔCT^ method, and all of the samples were analyzed independently in triplicate. Statistical analysis was conducted with GraphPad Prism 7.0. The primers used in this study are listed in Supplementary Table S2.

## 3. Results

### 3.1. Identification of COL Genes in H. citrina

**Figure 1:**
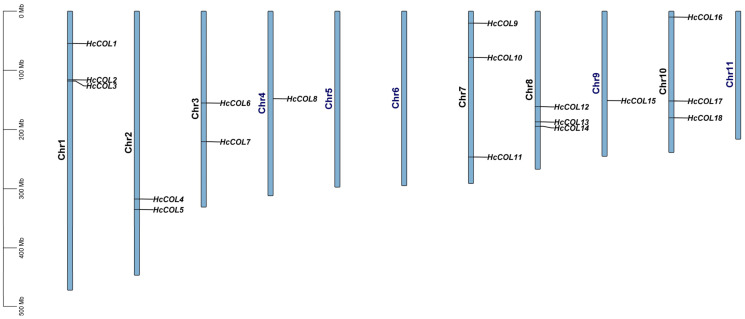
The distribution of 18 COL genes on 11 chromosomes of *H. citrina*.

The COL gene in *H. citrina* was identified on the basis of the COL gene family in *A. thaliana*, *O. sativa*, and *P. equestris*, which belong to the same order as *H. citrina*. Eighteen HcCOL genes were identified in the *H. citrina* genome, named *HcCOL1*-*HcCOL18* based on their chromosomal locations, encoding 19 HcCOL proteins, of which *HcCOL17* encodes two different HcCOL proteins, namely HcCOL17a and HcCOL17b (Figure 1). These genes were distributed across eight chromosomes in the genome. The physical and chemical properties of all the members were analyzed and predicted (Table 1). The lengths of the HcCOL genes ranged from 1040 to 18,306 bp, the CDS lengths ranged from 735 to 1545 bp, and the amino acid numbers ranged from 244 to 514. The molecular weights varied between 27.4 and 57.4 kDa, and the theoretical isoelectric point of the proteins ranged from 4.85 to 8.87, with 17 proteins predicted to be acidic proteins (pI < 7). Furthermore, all 19 HcCOL proteins were predicted to be localized in the nucleus.

### 3.2. Gene Structure and Protein Conserved Domain Analyses

**Figure 2:**
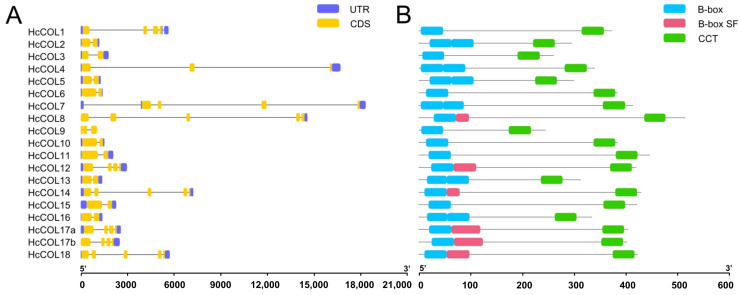
Structures of HcCOL genes and HcCOL proteins. (**A**) The structure of the HcCOL genes, with the blue box and yellow box representing the noncoding region (UTR) and coding sequence (CDS), respectively, and the black line representing the intron region. (**B**) The distribution of structural domains in the HcCOL proteins. The B-box, B-box SF, and CCT are represented by the cyan, pink, and green boxes, respectively.

**Figure 3:**
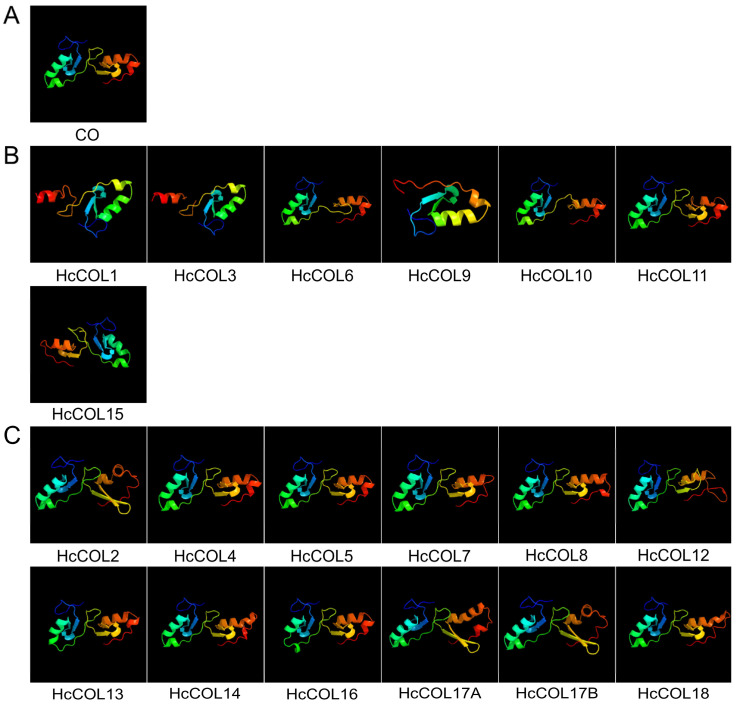
Predicted three-dimensional structures of the B-box domains of the CO and HcCOL proteins. (**A**) Two B-box domains from AtCO. (**B**) HcCOL protein containing only one B-box domain. (**C**) HcCOL protein containing two B-box domains (showing some HcCOL proteins with the second B-box structural domain as B-box SF).

The HcCOL gene contained 2–5 exons and 1–4 introns (Figure 2A). All HcCOL proteins conformed to the sequence features of the COL family and contained B-box and CCT domains. Among these HcCOL proteins, HcCOL1, HcCOL3, HcCOL6, HcCOL9, HcCOL10, HcCOL11, and HcCOL15 contained one B-box domain and one CCT domain, and HcCOL2, HcCOL4, HcCOL5, HcCOL7, HcCOL8, HcCOL12, HcCOL13, HcCOL14, HcCOL16, HcCOL17a, HcCOL17b, and HcCOL18 contained two B-box domains and one CCT domain (Figure 2B). In addition, the three-dimensional structure of the conserved B-box domain of CO and 19 HcCOL proteins was predicted using Phyre2 protein homology modeling technology (Figure 3). Figure 3B shows the HcCOL proteins with only one B-box domain, and the HcCOL proteins containing two B-box domains (Figure 3C) contained a B-box domain similar to that of CO (Figure 3A), with HcCOL4 and HcCOL5 having the highest similarity to the B-box domain of CO.

### 3.3. Sequence Alignment and Phylogenetic Analysis

**Figure 4:**
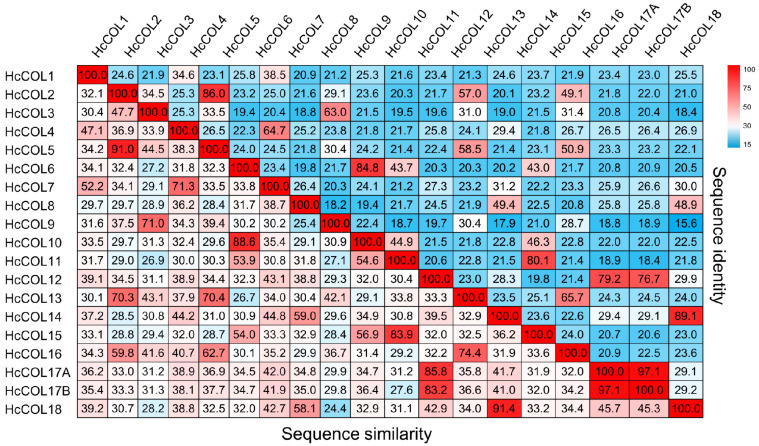
Sequence identities and similarities (%) between HcCOL proteins.

**Figure 5:**
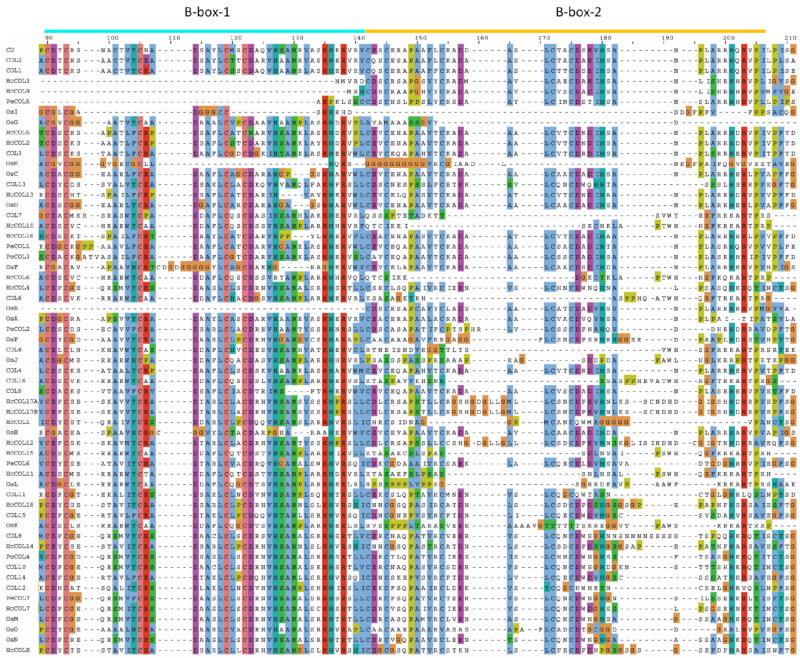
Comparison of the B-box domains of COL in *A*. *thaliana*, *O*. *sativa*, *P*. *equestris*, and *H*. *citrina*. The B-box1 and B-box2 domains are indicated by short lines in cyan and yellow, respectively, at the top of the sequences.

**Figure 6:**
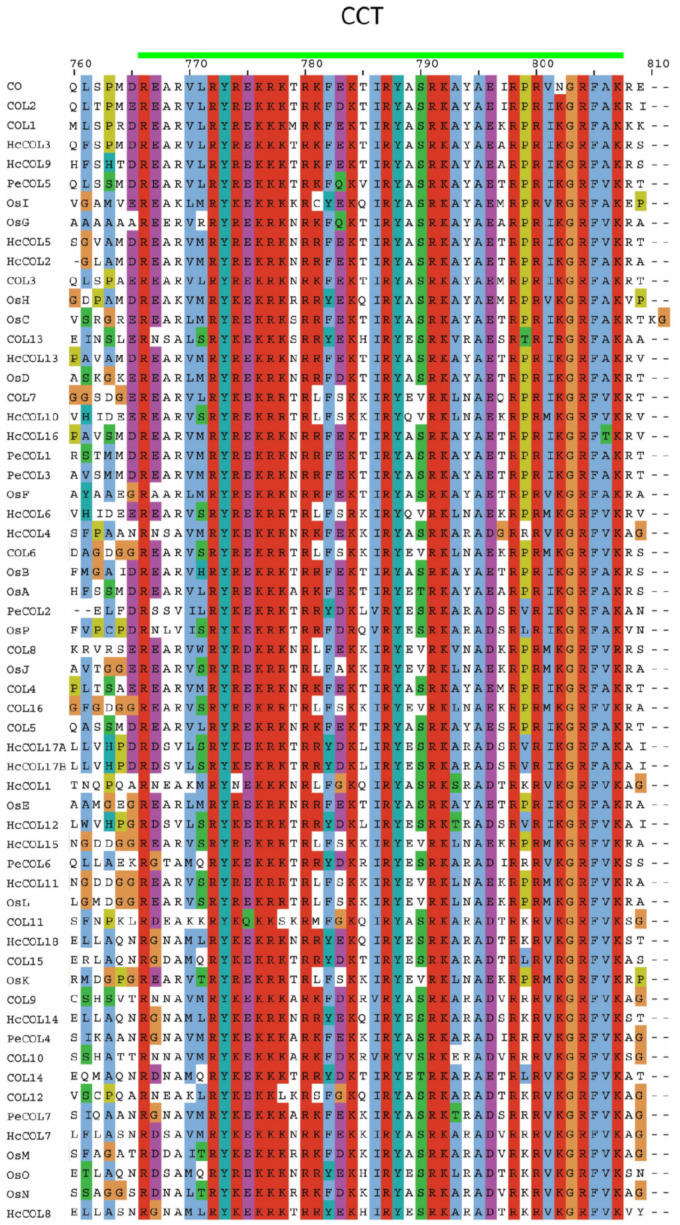
Comparison of the CCT domains of COL in *A*. *thaliana*, *O*. *sativa*, *P*. *equestris*, and *H*. *citrina*. The CCT domains are indicated by short lines in green at the top of the sequences.

**Figure 7:**
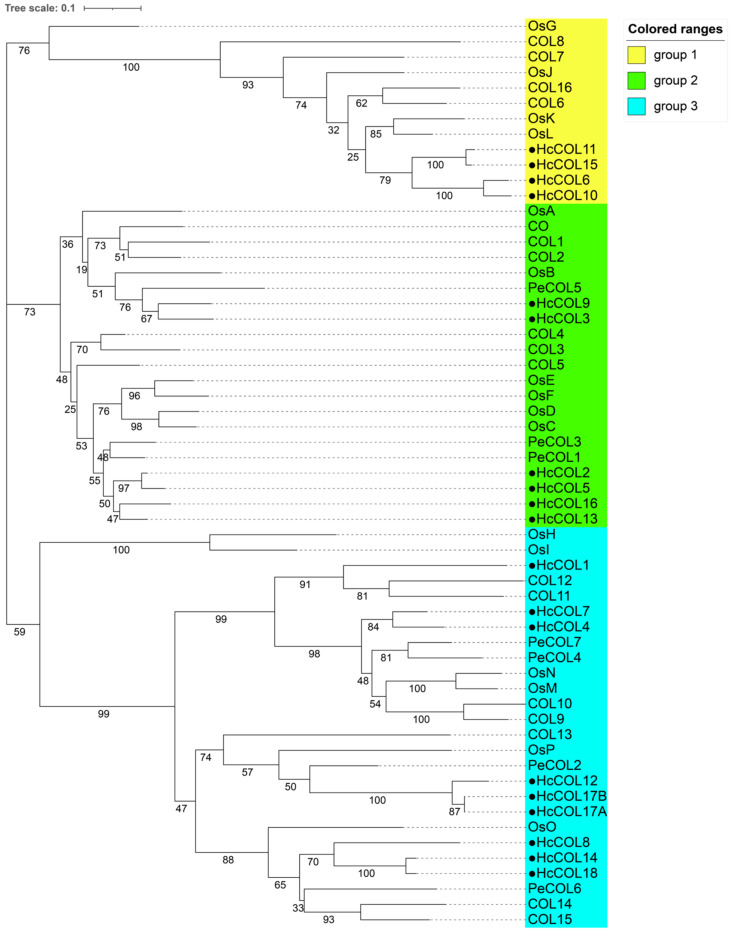
Phylogenetic tree of COL proteins from four plant species. The neighbor-joining method was used to conduct the phylogenetic analysis of COL proteins from *A*. *thaliana* (COL), *O*. *sativa* (Os), *P*. *equestris* (Pe), and *H*. *citrina* (Hc).

The pairwise alignment of 19 HcCOL amino acid sequences showed that the sequence similarity between HcCOLs ranged from 24.4% to 97.1%, and the sequence identity ranged from 15.6% to 97.1% (Figure 4). Among them, HcCOL17A and HcCOL17B had the highest similarity and identity, while HcCOL9 and HcCOL18 had the lowest similarity and identity. To further characterize the HcCOL proteins, multiple sequence alignments were performed on the amino acid sequences of COL proteins from *A. thaliana*, *O. sativa*, *P. equestris*, and *H. citrina* (Figure 5 and Figure 6). The results showed that the B-box and CCT domains in HcCOL were highly conserved. Among proteins containing one B-box, HcCOL1, HcCOL6, HcCOL10, HcCOL11, and HcCOL15 lacked the second B-box, while HcCOL3 and HcCOL9 lacked the first B-box. Among proteins containing two B-boxes, the second B-box in HcCOL8, HcCOL12, HcCOL14, HcCOL17A, HcCOL17B, and HcCOL18 was a B-box SF. To analyze the evolutionary relationships of COL proteins, a phylogenetic tree was constructed using the *A. thaliana*, *O. sativa*, *P. equestris*, and *H. citrina* COL proteins (Figure 7). The results showed that all 59 COL proteins were distributed on three branches, these three subgroups of which included four, six, and nine HcCOL members, respectively.

### 3.4. Collinearity Analysis within H. citrina and Among Different Species

**Figure 8:**
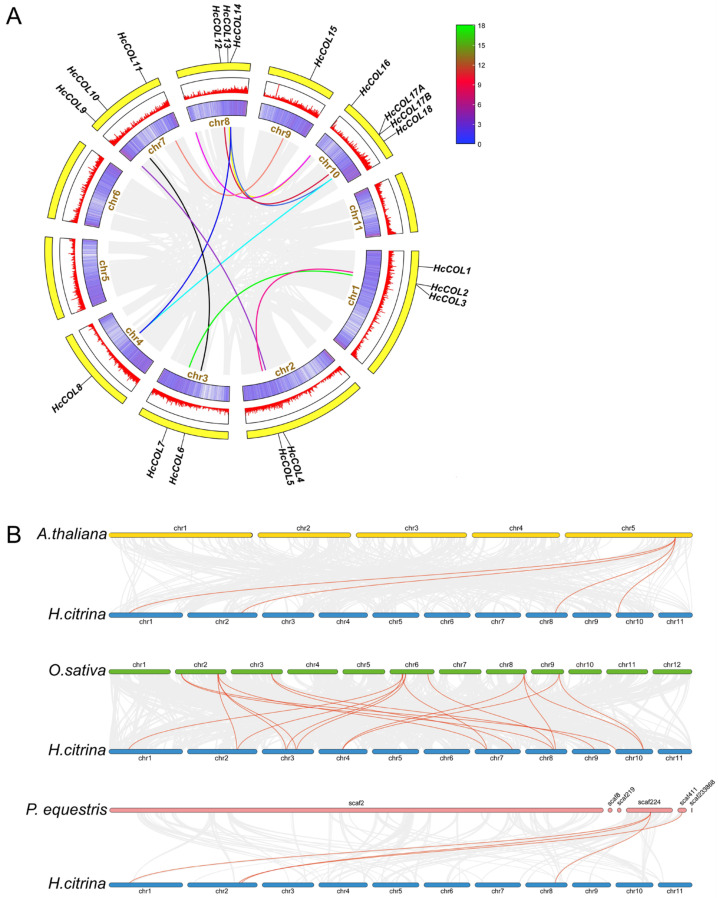
Synteny analysis of the COL gene family. (**A**) Schematic representation of the interchromosomal relationships between the HcCOL genes in the *H*. *citrina* genome. The colored lines connect the collinear gene pairs, and the gray lines indicate the syntenic blocks in the *H*. *citrina* genome. (**B**) Synteny analysis of the COL genes between *H*. *citrina* and 3 representative species. Red lines highlight the colinear gene pair, while gray lines indicate the syntenic blocks within the *H*. *citrina* and other plant genomes.

Analysis of genome-wide duplication is highly important for understanding the origin, evolution, and genome expansion of species. To investigate the evolutionary character of the HcCOL gene family, we analyzed the collinearity relationships between the HcCOL gene family and detected 11 duplication events, all of which were segmental duplications (Figure 8A), indicating that segmental duplication is the main expansion mode of the HcCOL gene family. We also analyzed the pairwise relationships of HcCOLs from *A. thaliana*, *O. sativa*, and *P. equestris* and detected 4 collinear gene pairs between *A. thaliana* and *H. citrina*, 19 pairs between *O. sativa* and *H. citrina*, and 4 pairs between *P. equestris* and *H. citrina* (Figure 8B). Although *H. citrina* and *P. equestris* belong to the Asparagales order, the COL genes have low homogeneity and may have evolved in different evolutionary directions. The COL genes in *H. citrina* and *O. sativa* have high homology and may have the same evolutionary direction. The Ka/Ks values of all duplicated COL gene pairs were calculated and found to be <1 for 23 gene pairs (Supplementary Table S3), indicating that the gene pairs underwent purifying (negative) selection during evolution.

### 3.5. Putative Cis-Acting Element and Functional Annotation Analysis

**Figure 9:**
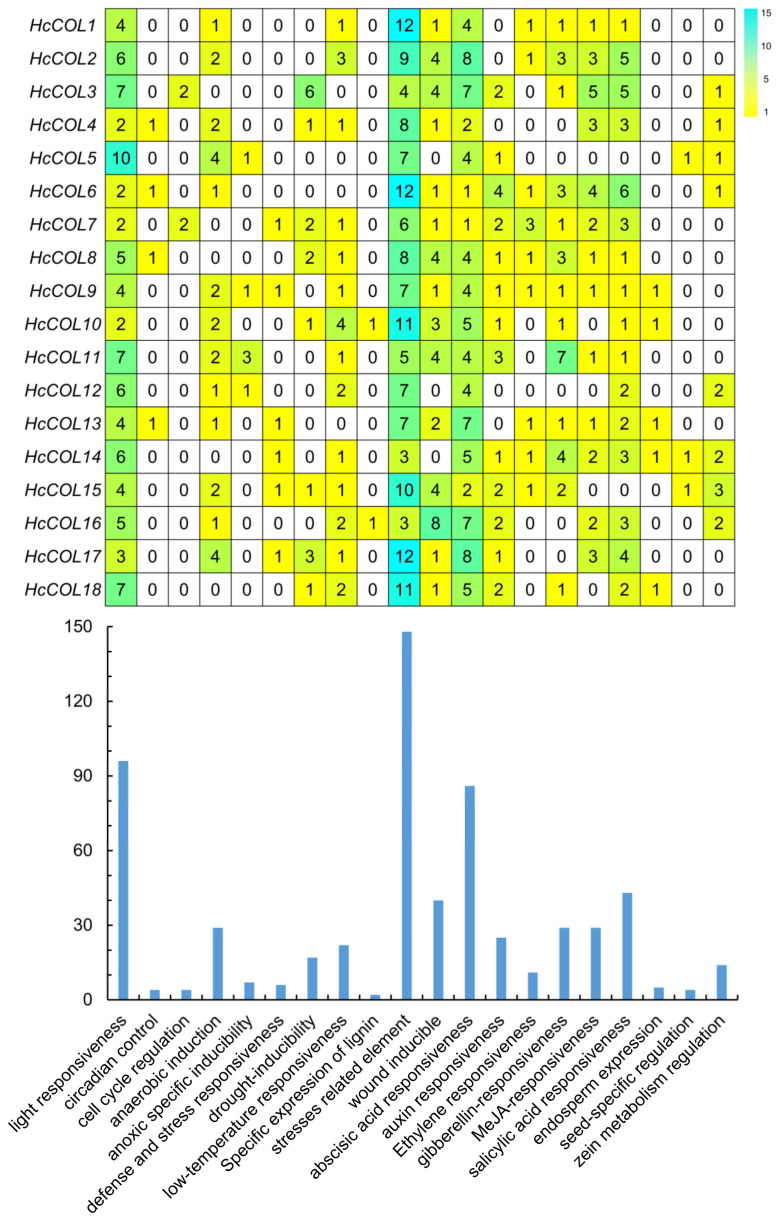
Analysis of *cis*-elements within the promoters of HcCOL gene family members.

The *cis*-acting elements of genes participate in gene regulation by interacting with corresponding *trans*-regulatory factors. Analyzing the *cis*-acting elements of a gene can provide valuable insight into the regulatory mechanism for its expression. The *cis*-elements of the promoter region of the HcCOL gene in *H. citrina* were identified (Figure 9). All 18 HcCOL gene promoters contained many light-responsive elements, consistent with their function as major regulators of the photoperiod pathway, four of which were involved in circadian control. In addition to *HcCOL3*, *HcCOL5*, *HcCOL6*, and *HcCOL13*, the remaining 14 HcCOL gene promoters contained low-temperature-responsive elements. The *HcCOL3*, *HcCOL4*, *HcCOL7*, *HcCOL8*, *HcCOL10*, *HcCOL15*, *HcCOL17*, and *HcCOL18* gene promoters contained drought-inducible elements, indicating that these genes may be involved in the abiotic stress response to low temperature and drought. The *HcCOL9*, *HcCOL10*, *HcCOL13*, *HcCOL14*, and *HcCOL18* gene promoters contained endosperm expression elements, which may be involved in endosperm development. All HcCOL genes contained phytohormone-inducible elements, including many responsive elements, such as abscisic acid-responsive elements, gibberellin-responsive elements, auxin-responsive elements, MeJA-responsive elements, and ethylene-responsive elements, indicating that the HcCOL genes may be involved in plant hormone regulation pathways.

### 3.6. HcCOL Gene Expression Analysis of Four Phases of Flower Development in H. citrina

**Figure 10:**
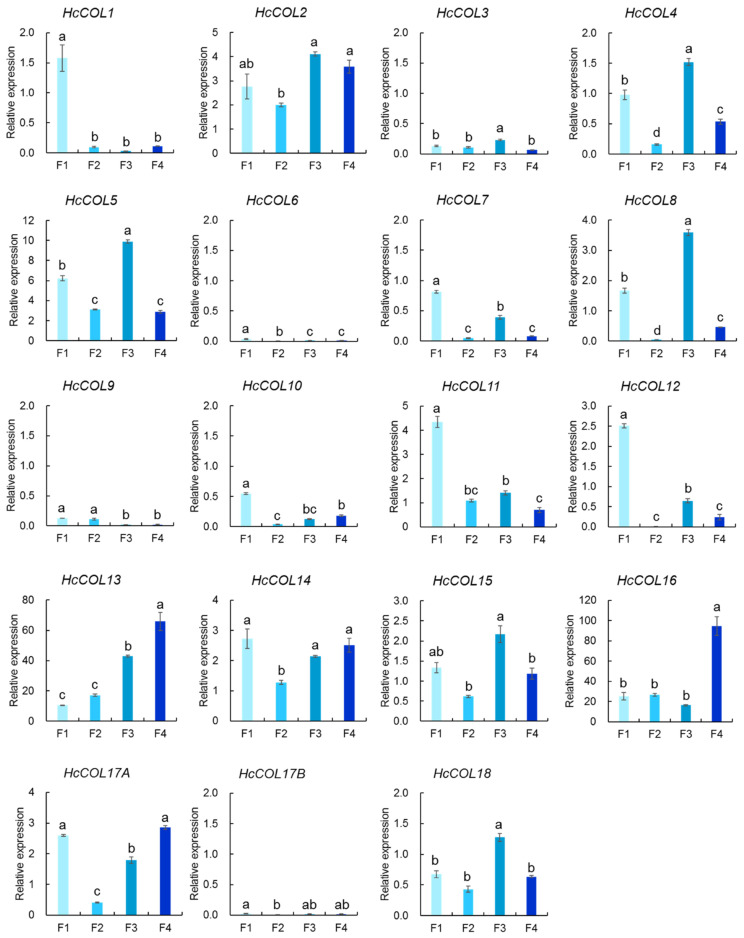
Expression levels of the HcCOL gene in F1 (young flower buds), F2 (flower buds), F3 (blooming flowers), and F4 (spent flowers). *AP4* was used as the internal reference gene. The values are the means ± SEMs of three biological replicates. The relative gene expression levels were calculated using the 2^−ΔΔCt^ method. The letters above the histogram represent significant differences at *p* < 0.05.

Previous studies have shown that COL family genes are synthesized in leaves and are involved mainly in flowering induction via the photoperiodic regulation pathway [39,40,41,42]. Recent research findings showed that the flowering time of ‘DatongHuanghua’ did not change after leaf shading but was significantly delayed when the flower buds were shaded, suggesting that flower buds are a critical component of the light response [43]. We speculated that HcCOL genes play a regulatory role after the flowering induction stage. To verify this, we measured the expression level of HcCOL in four phases of flower organ development (young flower buds, flower buds, blooming flowers, and spent flowers) (Figure 10). *HcCOL1*, *HcCOL11*, and *HcCOL12* were more highly expressed during the young flower bud phase than during the other phases; *HcCOL2*, *HcCOL4*, *HcCOL5*, *HcCOL8*, *HcCOL15*, and *HcCOL18* were more highly expressed during the blooming flower phase; and *HcCOL13*, *HcCOL14*, *HcCOL16*, and *HcCOL17A* were more highly expressed during the spent flower phase. Among them, the expressions of *HcCOL14* and *HcCOL16* in spent flowers exceeded that in young leaves. Based on these results, we surmised that the HcCOL gene may play a role in flower organ growth and flowering.

## 4. Discussion

Flowering is an important life process in plants that is regulated by the circadian clock in vivo, and light is one of the important timing-related factors in the input pathway of the circadian clock. As a core regulatory factor in the photoperiod pathway, CO integrates light signals and circadian clock signals to transmit flowering signals [44]. Many studies on this topic have been conducted in *A. thaliana* and *O. sativa*. *H. citrina* is a perennial herbaceous plant with a long vegetative phase that lasts approximately three years before flowering. *H. citrina* is a LD-type plant like *A. thaliana*, and COL genes likely play a similar role in both plants. However, research on the role of the COL gene in *H. citrina* flowering is still limited, and the specific function of COL genes in *H. citrina* needs to be verified through further research. The recently assembled *H. citrina* genome contains chromosome-level gene localization information [45], which enables comprehensive analysis of the COL gene family in *H. citrina*. In this study, 18 COL genes, encoding 19 HcCOL proteins, were identified in the *H. citrina* genome. All the HcCOL proteins contained B-box and CCT domains, with six proteins containing two B-box domains, seven proteins containing only one B-box domain, and six proteins containing one B-box domain and one B-box SF domain. Previous studies have shown that genes containing two B-box domains are involved in the photoperiodic signaling pathway or photoperiodic flowering pathway [46].

Members of the COL family in *A. thaliana*, *O. sativa*, and *P. equestris* were classified into three groups with *H. citrina,* based on phylogenetic analyses, which is similar to the grouping in *O. sativa* and *A. thaliana* [47]. The results of the synteny analysis indicate that segmental duplication is the main expansion mode of the HcCOL gene family. The highest number of homologous genes was detected between *H. citrina* and *O. sativa*, with *OsE*, *OsF*, *OsJ*, *OsK*, and *OsN* each having two homologous genes in *H. citrina* and *OsO* having three homologous genes in *H. citrina*, suggesting a close relationship between the COL genes of *H. citrina* and *O. sativa*. Although *H. citrina* and *P. equestris* belong to the same order, *Asparagales*, the number of homologous genes between them was the lowest, with *PeCOL3* having three homologous genes and *PeCOL4* having one homologous gene in *H. citrina*. This result is presumably related to the smaller number of COL gene family members in *P. equestris*. The Ka/Ks values of all 23 COL gene pairs (some homologous gene pairs have too much sequence variation to yield Ka/Ks values) were less than 1, indicating that the COL genes underwent purifying selection during evolution.

The edible part of *H. citrina* is the flower bud, and the flowering time is not more than 24 h. Recent studies have shown that flower buds are important for the response to light in the photoperiod pathway of *H. citrina* [39]. To understand whether the HcCOL genes play a role in the growth, flowering, and senescence of flowers, we used flower organs at four growth stages as research materials to detect the mRNA expression levels of HcCOL genes. *HcCOL1*, *HcCOL11*, and *HcCOL12* exhibited higher expression levels during the early bud stage, suggesting their potential involvement in the growth and development of floral organs. On the other hand, *HcCOL2*, *HcCOL4*, *HcCOL5*, *HcCOL8*, *HcCOL15*, and *HcCOL18* displayed relatively high expression levels during the flowering stage, indicating that they may promote the flowering process by increasing their expression in *H. citrina* flowers. Additionally, *HcCOL13*, *HcCOL14*, *HcCOL16*, and *HcCOL17A* exhibited increased expression levels during the spent flower stage, suggesting their potential involvement in the senescence of floral organs.

Our research revealed that all the HcCOL gene promoter regions contained light-responsive elements, indicating that HcCOL genes are likely involved in the photoperiodic regulatory pathway. Additionally, some gene promoter regions contained elements that are inducible by cold and/or drought, suggesting their potential involvement in cold and/or drought stress responses. Furthermore, all the gene promoter regions contained plant hormone-inducible elements, indicating that the expression of HcCOL genes may be associated with phytohormone regulatory pathways. In this study, the HcCOL gene family was systematically bioinformatically analyzed, laying the foundation for subsequent research on the function of HcCOL genes, and it is worthwhile to conduct further in-depth studies on the specific functions of HcCOL genes.

## Figures and Tables

**Table 1:** HcCOL gene sequence information and physicochemical properties of the proteins.

Gene	Transcript ID	Length of ORF (bp)	Length of cDNA (bp)	AA	Chromosome Position	MW (kDa)	pI	Predicted Location
*HcCOL1*	*HHC001023.1*	5639	1119	372	Chr1	40,466.48	5.24	Nucleus
*HcCOL2*	*HHC002209.1*	1193	888	295	Chr1	32,518.03	6.02	Nucleus
*HcCOL3*	*HHC002254.1*	1791	783	260	Chr1	29,533.84	5.14	Nucleus
*HcCOL4*	*HHC009483.1*	16,688	1020	339	Chr2	37,039.32	8.87	Nucleus
*HcCOL5*	*HHC009754.1*	1282	900	299	Chr2	32,795.27	6.33	Nucleus
*HcCOL6*	*HHC012875.1*	1405	1152	383	Chr3	43,535.87	5.77	Nucleus
*HcCOL7*	*HHC013899.1*	18,306	1242	413	Chr3	45,350.19	5.07	Nucleus
*HcCOL8*	*HHC019369.2*	14,562	1545	514	Chr4	57,358.38	7.02	Nucleus
*HcCOL9*	*HHC030027.1*	1040	735	244	Chr7	27,408.75	6.00	Nucleus
*HcCOL10*	*HHC030937.1*	1520	1152	383	Chr7	43,026.35	5.67	Nucleus
*HcCOL11*	*HHC032260.1*	2090	1341	446	Chr7	49,662.55	5.00	Nucleus
*HcCOL12*	*HHC034570.1*	2966	1260	419	Chr8	47,005.43	4.85	Nucleus
*HcCOL13*	*HHC034944.1*	1383	939	312	Chr8	34,296.95	5.84	Nucleus
*HcCOL14*	*HHC035073.1*	7230	1290	429	Chr8	46,963.08	5.58	Nucleus
*HcCOL15*	*HHC038535.1*	2279	1266	421	Chr9	46,404.69	5.07	Nucleus
*HcCOL16*	*HHC040621.1*	1391	1005	334	Chr10	36,251.84	7.98	Nucleus
*HcCOL17a*	*HHC041944.1*	2697	1215	404	Chr10	44,434.74	5.47	Nucleus
*HcCOL17b*	*HHC041944.2*	2697	1209	402	Chr10	44,224.5	5.40	Nucleus
*HcCOL18*	*HHC042453.1*	5721	1269	422	Chr10	46,607.94	5.32	Nucleus

## Data Availability

Data is contained within the article and Supplementary Materials.

## Supplementary Materials

The following supporting information can be downloaded at: https://www.mdpi.com/article/10.3390/cimb46080503/s1, Table S1: The COL genes in four plants; Table S2: Primers used in this study; Table S3: Ka/Ks analysis for duplicated COL gene pairs.
